# Regional Disparities in the Decline of Anemia and Remaining Challenges among Children in Tanzania: Analyses of the Tanzania Demographic and Health Survey 2004–2015

**DOI:** 10.3390/ijerph17103492

**Published:** 2020-05-17

**Authors:** Bruno F. Sunguya, Si Zhu, Linda Simon Paulo, Bupe Ntoga, Fatma Abdallah, Vincent Assey, Rose Mpembeni, Jiayan Huang

**Affiliations:** 1 School of Public Health and Social Sciences, Muhimbili University of Health and Allied Sciences, Dar es salaam 11103, Tanzania; sunguya@gmail.com (B.F.S.); linda.p.simon@gmail.com (L.S.P.); rcmpembeni@gmail.com (R.M.); 2 School of Public Health, Fudan University, Shanghai 200032, China; zhus17@fudan.edu.cn; 3 Global Health Institute, Fudan University, Shanghai 200032, China; 4 Key Laboratory of Health Technology Assessment, National Health Commission, Shanghai 200032, China; 5 Tanzania Food and Nutrition Center, Dar es salaam 11101, Tanzania; bntoga@yahoo.com (B.N.); drfatmak@yahoo.com (F.A.); vdassey@gmail.com (V.A.)

**Keywords:** anemia, children, Tanzania, demographic dividend, Demographic and Health Survey, nutritional actions

## Abstract

The burden of child anemia is on the decline globally but remains prevalent in low- and middle-income countries, including Tanzania. Evidence suggests regional variation and a slow pace of decline even in areas with high food production. The factors behind such decline and remaining challenges behind child anemia remain understudied in Tanzania. This secondary data analysis utilized data including 7361 and 7828 children from the Tanzania Demographic and Health Surveys (TDHS) conducted in 2004–2005 and 2015–2016 separately to examine the decline of child anemia and regional variation thereof. We used a geographic information system (GIS) to visualize the changes and differences between regions and the two study periods, and used regression analyses to examine the recent determinants of child anemia. Anemia has declined among children under five in Tanzania by 42% over a one-decade period, but remained high in relatively high food-producing regions. The risk of anemia is still higher among boys compared to girls (AOR = 1.39, *p* = 0.005), 41% higher among children lived in households with more than three under-five children compared to those households with only one child (*p* = 0.002); lower among children whose mothers were educated (*p* < 0.001) or had first given birth when aged over 25 (*p* = 0.033); and 34% less among children in the wealthiest households (*p* < 0.001). Efforts are needed to address social determinants of health, especially targeting women’s empowerment through decreasing the number of children and encouraging child spacing, and poverty reduction, particularly in high food producing regions.

## 1. Introduction

The global burden of anemia declined from 40.2% in the 1990s to 32.9% in 2010, but the decline varied widely across countries [1]. Despite such slow decline over the period of twenty years, the years lived with disability (YLD) to anemia increased from 65.4 to 68.5. The highest burden of anemia was noted in the Eastern Africa region. Anemia affects vulnerable populations including children under the age of five years disproportionately compared to the general population [2,3,4,5]. The long-term effects caused by anemia among children is a challenge to national development. Addressing this burden is therefore necessary to reverse the long-term consequences for sustainable development [6].

Tanzania, like other low- and middle-income countries, suffers from various forms of undernutrition, anemia being one of them. Anemia is prevalent in 59% of children under the age of five in the country. If left unaddressed, such an unprecedented burden will jeopardize the ongoing efforts to attain the nation’s industrialization vision and middle-income level status. Nutritional anemia is one of the important anemia types in Tanzania. It is mostly caused by the consumption of food with inadequate iron sources. Other important causes are diseases such as malaria and worm infestations which lead to a loss of iron through hemolysis and blood loss. The latter have declined significantly owing specific interventions in malaria and soil transmission helminths control. Although anemia remains high in Tanzania, it seems to follow the patterns of other nutritional challenges such as stunting, underweight, and wasting, which are uniquely higher even in food producing regions [7,8,9]. Previous evidence suggests that regions with high food production have higher proportions of peasant farmers engaging in monoculture and spending many hours in farming activities, affecting childcare. Owing to poor feeding practices, poor hygienic practices, lack of knowledge on nutritional foods, and poor access to health services owing to the nature of the economic activities of caregivers in these areas, children succumb to poor nutritional status including anemia [8,10]. These regions include Mbeya, Rukwa and Shinyanga, with the prevalence of anemia at 55%, 53% and 71%, respectively [8].

In addressing the burden of anemia and other nutritional disorders, the government of Tanzania has been implementing a five-year national multisectoral nutrition action plan (NMNAP) [11]. This action plan enabled the country’s nutrition response coordinating arm—the Tanzania Food and Nutrition Center (TFNC)—to identify key areas and targets to be reached by 2021. They included a 30% reduction in the prevalence of child anemia from the 2015 prevalence of 58%. Such an ambitious target calls for tailored nutrition-specific and sensitive interventions owing to regional and causal differences [10,12,13,14]. Tanzania is not among countries facing regular food shortages; however, more than 70% of the population live in rural areas practicing monoculture food production [15]. Such an economical model limits feeding practices to a narrow dietary diversity leading to inadequate intake of micronutrients including iron [16,17]. Over the years, the national efforts to address undernutrition through improving access to health, hygienic interventions, and nutrition counseling have resulted in a decline in all types of undernutrition [8], but with wide variations within and between the regions, and an overall unprecedented burden.

A number of interventions have been conducted in regions to address undernutrition. Such interventions may also be responsible for the evidenced disparities [8,18,19,20], but data have not been analyzed to explain the trends within the regions. Moreover, although efforts to address the remaining burden call for tailored interventions, evidence on the remaining determinants of child anemia remain understudied. In addressing these evidence gaps, we analyzed the nationally representative surveys to first examine trends of child anemia in Tanzania during the last 10 years. We also used geographic information system (GIS) mapping to show the regional disparity in the decline in anemia. Second, we examined changes in the determinants associated with child anemia and used the recent survey to examine the remaining determinants of anemia among children under five in Tanzania.

## 2. Materials and Methods 

### 2.1. Study Design 

This secondary data analysis utilized the last two Tanzania Demographic and Health Surveys (TDHS) conducted in 2004–2005 [18] and 2015–2016 [8]. These nationally representative surveys employ cross sectional study designs to examine and follow up maternal and child health indicators. They are conducted every four years by the National Bureau of Statistics (NBS) and Zanzibar Bureau of Statistics (ZBS) under the USAID funding and technical leadership of MEASURES. The available data included a total of 15,189 children. Of them, 7361 were from 2004–2005 and 7828 from the 2015–2016 TDHS survey. We used GIS to map the changes between the two study periods.

### 2.2. Data Analysis 

Data were analyzed using SPSS version 22 software (IBM, Armonk, NY, USA). The dependent variable was child anemia, defined as hemoglobin (Hb) concentration less than 11 g/dL. This was categorized as mild anemia (Hb 10–11 g/dL), moderate anemia (Hb 7–9.9 g/dL) and severe anemia (Hb,7 g/dL). We used Pearson’s chi-square tests to examine the differences in the severity of anemia and other independent variables between the two periods. Such independent variables included demographic, household and feeding characteristics. For geographical variation, we examined the regional differences in relation to the two study periods.

We conducted a three-level hierarchical logistic regression model to examine the changes in child anemia in Tanzania over the two survey points after adjusting for confounders and covariates for simultaneous changes over time. In the first model, we only included the survey phase. In the second model, we included caregivers’ characteristics and household characteristics related factors and the factor from the first model which had a *p*-value < 0.2. In the final (third) model, we included child characteristics-related factors, and factors from the first and second models which had a *p*-value < 0.2. We also conducted a two-level hierarchical logistic regression analysis to examine the remaining factors associated with anemia for policy implications using TDHS 2015–2016. Finally, we used the GIS to display the disparity of the decline rate of child anemia prevalence within 30 regions. We presented the differences in a color-coded map to display such changes.

## 3. Results

### 3.1. Changing Landscape in Child Anemia in Tanzania

The magnitude of child anemia in Tanzania has declined significantly from 70.9% in 2004 to 58.7% in 2015 (*p* < 0.001). Except for mild forms of anemia, there is a statistically significant decline in moderate anemia from 42.8% to 30.3% (*p* < 0.001), and severe anemia from 4.1% to 1.7% (*p* < 0.001) in the past ten years (Table 1).

Although there has been a general decline in anemia over the ten years, there is also a notable variation across age categories. For example, the magnitude of anemia among 0-11-month-old children increased from 72.6% in 2004 to 79.2% in 2015 (Table 2). In comparison to children in urban areas, those residing in rural areas continued to succumb to higher burdens of anemia (*p* < 0.001). However, there is also a notable decline from 72.1% in 2005 to 60.0% in 2015 (Table 2). The magnitude of anemia increased with the size of the family, although the difference between years did not reach a statistical significance level. The number of children increased the likelihood of a child to be anemic, and this was consistent over the one decade under follow up (*p* = 0.004). The education level of a caregiver remained inversely proportional to child anemia. The children of more educated mothers were more likely to have lower magnitudes of anemia (*p* < 0.001). Caregivers in married relationships had lower magnitudes of anemic children compared to other marital statuses. There was a significant decline in such magnitude from 70.6% to 58.9% over the decade under survey (*p* < 0.001). Magnitudes of anemia also varied with wealth index. The higher wealth quintile was associated with a lower magnitude and drastic change in the magnitude of anemia over the ten years under survey (*p* < 0.001). 

Comparison of the magnitude of anemia with regard to dietary diversity was made for the 2015 dataset alone (not shown in the table). This is because similar data were not available for the 2004 dataset. Anemia seems to be more common among children with low dietary diversity score (67.3%) compared to those with higher dietary diversity score (66.3%). However, such difference did not reach a statistically significant difference. Moreover, only 84 children were reported to be given iron supplements. There were no statistically significant differences in terms of anemia prevalence among them compared to their counterparts who were not being provided with iron supplements, owing to a small sample size. The prevalence of anemia among children who were targeted by anthelmintic was 10% lower compared to among their counterparts who were not given such medications (55.7% vs. 62.3%).

### 3.2. Adjusted Differences in the Burden of Anemia between 2005 and 2016

We combined data of 2004/2005 and 2015/2016 to examine the changing landscapes of anemia while adjusting for important confounders and covariates found in the previous analyses. Table 3 below shows the significant improvement in anemia among children under five after adjusting for such confounders and covariates. Children under five in the 2015–2016 survey were 42% less likely to be anemic compared to their counterparts in 2004–2005 survey (p < 0.001) after adjusting for other covariates and confounders. In these combined datasets, anemia was associated with family size, number of children in a household, mother’s education level and wealth index. As expected, children with stunting were more likely to be anemic after adjusting for other confounders and covariates. 

**Figure 1:**
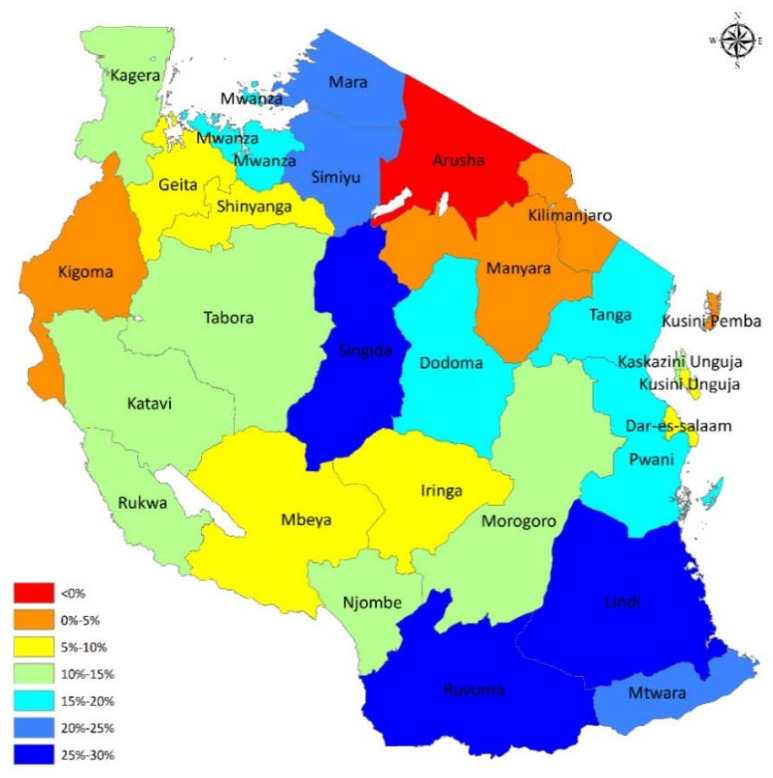
Geographic information system (GIS) mapping of the magnitude change of the burden of child anemia.

Although with wide variation, the magnitude of child anemia improved in all but one region. All but one region observed a decline in the prevalence of anemia. The prevalence of anemia increased from 53.4% in 2004 to 59.0% in 2015 in the Arusha region. Regions with higher magnitudes of anemia observed a more significant decline compared to those with relatively lower magnitudes at the baseline (2004). Such regions include Lindi, Ruvuma, Singida, Mtwara, and Simiyu. Figure 1 shows the changes in magnitudes of anemia among children over the ten-year period. The GIS mapping suggests a more than 25% difference in the magnitude of child anemia between the two reference periods in three regions (Singida, Lindi, and Ruvuma). No or little difference of less than 5% was observed in Kigoma, Arusha, and Kilimanjaro, and a difference of between 5% and 10% found in the lake zone (Geita and Shinyanga), southern highlands (Mbeya and Iringa), Dar es salaam, and Kusini Unguja regions. The rest of the regions observed a moderate difference of between 10% and 25%. This analysis suggests areas with high food production (southern highlands) have a modest change in the burden of anemia. Moreover, there is no trend that suggests regional similarity according to weather, food productivity, or urbanization, as the changes are not in any particular pattern. 

### 3.3. The Remaining Factors Associated with Child Anemia in Tanzania

We analyzed the most recent available data, 2015–2016, to examine the remaining factors associated with child anemia in Tanzania. Table 4 shows analyses of two-level logistic regression analyses to examine the independent factors associated with anemia after adjusting for confounders and co-variates. A child in a household with more than three under-fives was more likely to have anemia (AOR = 1.41, *p* = 0.002). Compared to children whose mothers were aged under 15 at first birth, those whose mothers were aged above 25 were less likely to have anemia (AOR = 0.68, *p* = 0.033). Similarly, children whose mothers were educated were less likely to have anemia. Similarly, after adjusting for covariates and confounders, households in the higher and highest wealth quintile were less likely to have an anemic child (AOR = 0.71, *p* < 0.001, AOR = 0.66, *p* < 0.001, respectively). Child anemia declined with child age, although the association did not reach a statistically significant difference. Male children were more likely to have anemia (AOR 1.39, *p* = 0.005). Finally, children born with normal or high birthweight were 40% less likely to have anemia compared to children with low birthweight (*p* = 0.026).

## 4. Discussion

The burden of child anemia has significantly declined over the past decade in Tanzania, but remains unacceptably high [8]. Our analyses revealed a 42% decline in the burden of anemia from the 2004 level (71%) to the 2015 level (59%), suggesting a significant decline in a such a devastating condition [21,22,23,24]. This was also attributed to a significant decline in both moderate and severe forms of anemia. During this period, there was also notable improvement in other social determinants of health, including education attainment and women empowerment, that might explain improvement of this nutritional indicator. Despite the notable decline, anemia remains prevalent in more than one in two children and will therefore negatively impact their future, rendering them incapable of attaining their intellectual capacity [23]. This unprecedented burden calls for immediate multisectoral actions using available resources to sustain the gains.

Our analyses also noted the regional variation in the burden of anemia in Tanzania. Uniquely to Tanzania, regions with seemingly high food productivity present with higher magnitudes of children anemia compared to the national average [8]. Like in other low- and middle-income countries, the most important form of anemia in Tanzania is of the iron deficiency type. Also called nutritional anemia, iron deficiency anemia is caused by either suboptimal consumption of iron rich foods or infections such as malaria and infestations caused by parasitic worms causing a loss of iron. With effective interventions addressing malaria and parasitic works, improved health seeking behavior, and access to care, the remaining important cause of nutritional anemia is poor feeding practices. Although the difference did not reach a statistically significant level, analysis of the 2015 database revealed a slightly higher burden of anemia among children fed with less diverse foods compared to their counterparts. With more than 70% of the population residing in rural Tanzania, and their main economic activity being small scale farming and husbandry [15,25], they consume mainly from their main domestic food production. In regions with high food productivity, most peasants rely on monoculture, and in particular cereal and to a larger extent legumes [19,26]. This results in food insecurity and therefore poor feeding patterns, leading to nutritional inadequacy [27,28]. Apart from the narrow range in dietary diversity [29], such farming populations are subjected to seasonal food insecurity owing to cyclic food availability and rain uncertainties [30]. Therefore, despite their capacity to feed the nation, they remain with a little to feed their families and, in particular, nutritional and iron dense foods for their children. Similar to anemia, these regions have high magnitudes of stunting [8]. Nutritional anemia and stunting are both chronic forms of undernutrition and stem from similar determinants and causes [31,32]. 

Our results also show that boys have a high prevalence of anemia. Traditionally, boys in Tanzania families are free to play around but girls need to stay at home to take care of siblings. In other words, girls have more access to food that is needed for growth and development while boys expend large amounts of energy which should have been channeled into increasing growth. Our results were further solidified in analyses of the entire datasets where anemia was associated with stunting. Such findings, however, were not significant when the 2015 dataset was analyzed separately. Moreover, there was no significant difference between rural and urban areas in the adjusted analyses, calling for more efforts streamlined to address other social determinants of health that may cut across the geographical divides. A decline in the number of children would also decrease the number of people in the household, which was also associated with anemia. Through this population divided, the households and communities would be wealthier, which strongly correlated with a decline in anemia after addressing other confounders.

Although there was a 42% decline from the 2004 level after adjusting for other changes, some regions have had a more surprising decline in anemia than others in absolute figures and unadjusted percentages. Three regions (two in the southern zone and one in the central zone) had between 25% and 30% decline. Ruvuma, Lindi and Singida have different demographic, geographic, and economic characteristics—Ruvuma is among the food-producing giants in Tanzania, while Lindi and Singida are not. Four regions, namely Kigoma, Arusha, Kusini Pemba, and Kilimanjaro, had poor trends of anemia. In particular, Arusha, which is also a tourism hub of the country, with good weather, and accessible in all directions by roads, and therefore food access from other regions observed an increase in anemia by five percentage points. The rural areas of Arusha are characterized by varied sizes of animal husbandry, with the vast population occupied by the “Maasai tribe”, who are nomads and succumb to long spells of food insecurity, leading to various chronic forms of undernutrition, including anemia [33,34,35]. Poor access to health services and interventions including safe drinking water and sanitation could be behind such an increase [33]. Efforts to mitigate such determinants might have been unsuccessful and more efforts tailored to such populations are needed. The Kilimanjaro region has a diverse population, but is also characterized by unhealthy feeding habits coupled by restrictive feeding beliefs [36]. Kigoma is also a region with a high burden and a low decline rate for child anemia. In Kigoma, the refugees and migrant populations from relatively unstable neighboring countries could be contributing to such unprecedented magnitude of anemia owing to their social and economic disadvantages. 

The national guidelines for the management of anemia (2019–2022) pledged a 30% reduction in the prevalence of anemia in children under five years of age from 58% to 40% in 2022. To achieve this ambitious target, owing to the slow pace of decline in the last decade, multisectoral efforts and lessons from regions with rapid decline are essential. Such lessons can be tailored to regions with high burden. Partners involved in the decline in anemia in regions that performed well and interventions that seemed to work can be carefully considered in regions with poor performance of anemia and other forms of undernutrition indicators. It is therefore necessary for them to provide guidance and technical expertise to address the local challenges and determinants thereof. 

## 5. Conclusions

In conclusion, Tanzania has observed a 42% decline in child anemia over the past decade after adjusting for other changes in the same period. However, the rate of decline is not adequate for the pace required to reach the national and global targets and varies widely between the regions. Notably, three regions (Lindi, Ruvuma and Singida) had an outstanding decline of above 25% and are on the right course for reaching the national target of 30%. Four other regions (Kigoma, Arusha, Kusini Pemba, and Kilimanjaro) observed a very slow or negative trend and more efforts are needed. Moreover, improvements in social determinants of health, including women empowerment through educating women and girls, smaller number of children per woman, smaller number of children and family members per households, and poverty, were important determinants for such decline. Going forward, it is important to address the remaining determinants of anemia. This can be achieved through strengthening the pace towards improvement of social determinants of health. Efforts should be specifically targeted at educating women and addressing child spacing. Targeting areas with high burden and food productivity will be essential through ensuring adequate nutritional education, alleviating seasonal food insecurity, access to health care and social services. Lessons from the successful models in the best performing regions will provide local solutions when tailored to poorly performing regions to attain the national and global sustainable development goals. 

## Figures and Tables

**Table 1:** The prevalence of anemia among children under 5 in Tanzania.

Anemia Status	2004/2005		2015/2016		*p*-Value
	N	%	n	%	
Normal	2141	29.1	3232	41.3	
Anemia status	5220	70.9	4596	58.7	<0.001
Mild anemia	1762	23.9	2091	26.7	0.834
Moderate anemia	3153	42.8	2369	30.3	<0.001
Severe anemia	305	4.1	136	1.7	<0.001
Total	7361		7828		

**Table 2:** Individual and household characteristics in relation to changing burden of anemia among children under five in Tanzania.

Variable	Anemia in 2004/2005		Anemia in 2015/2016		*p*-Value
	N	%	N	%	
**Age**					
0–11	1250	72.63	773	79.20	<0.001
12–23	1320	82.55	1515	73.62	
24–35	1094	74.07	968	57.24	
36–47	836	62.25	697	44.06	
48–59	720	58.92	642	42.24	
**Sex**					
Male	2629	71.34	2388	60.29	0.115
Female	2591	70.50	2208	57.10	
**Birthweight**					
Low	343	69.86	331	53.91	0.091
Normal or High	2194	69.32	2434	56.45	
**Type of residence**					
Rural	4322	72.13	3495	60.01	<0.001
Urban	898	65.60	1101	54.94	
**Family size**					
1–4	1226	68.99	990	55.62	0.009
5–9	3033	70.32	2666	57.89	
10+	961	75.61	940	65.14	
**Number of children under 5**					
1	1531	69.81	1480	55.66	0.004
2	2087	69.89	1779	57.41	
3	997	71.73	787	62.76	
>3	509	77.71	470	69.63	
**Mother’s age at first birth**					
<15	149	63.68	136	67.66	0.045
15–19	3270	72.01	2754	59.38	
20–24	1520	69.44	1428	58.45	
≥25	281	70.78	278	50.92	
**Education level (mother)**					
No education	1423	74.78	1129	66.45	<0.001
Primary	3566	69.54	2873	56.87	
Secondary	182	71.65	565	55.72	
Higher	49	65.33	29	45.31	
**Mother’s Marital Status**					
Married	4109	70.60	2911	58.94	<0.001
Living together	429	70.68	971	59.50	
Widowed/Divorced/Live Apart	472	72.84	496	57.34	
Never Married	210	73.43	218	55.61	
**Wealth index**					
Poorest	1293	77.06	1220	63.81	<0.001
Poorer	1138	73.94	1052	61.41	
Middle	1124	70.51	915	59.73	
Richer	993	67.87	756	53.28	
Richest	671	61.79	653	52.11	

**Table 3:** Decline in child anemia in relation to other factors using TDHS 2004–2005, TDHS 2015–2016.

Variable	N(%)	Model 1 ^a^		Model 2 ^b^		Model 3 ^c^	
		AOR(95% CI)	*p*-Value	AOR(95% CI)	*p*-Value	AOR(95% CI)	*p*-Value
**Survey year**							
2004–2005	7976(46)	1.00					
2015–2016	9520(54)	0.58(0.55,0.62)	<0.001				
**Household characteristics**							
**Type of residence**							
Urban	4099(23)			1.00			
Rural	13,397(77)			0.9(0.81,1.01)	0.064		
**Family size**							
1–4	4230(24)			1.00			
5–9	10,078(58)			1.02(0.93,1.11)	0.672		
10+	3188(18)			1.2(1.05,1.37)	0.007		
**Number of children under 5**							
1	5553(33)			1.00			
2	6790(40)			0.97(0.89,1.05)	0.442		
3	2993(18)			1.06(0.94,1.18)	0.337		
>3	1518(9)			1.26(1.07,1.49)	0.006		
**Mother’s age at first birth**							
<15	511(3)			1.00			
15–19	10,609(61)			1.17(0.95,1.44)	0.139		
20–24	5288(30)			1.13(0.92,1.40)	0.254		
≥25	1089(6)			1.07(0.84,1.37)	0.594		
**Mother’s education level**							
No education	4099(23)			1.00			
Primary	11,668(67)			0.81(0.74,0.88)	<0.001		
Secondary	1554(9)			1.01(0.87,1.18)	0.89		
Higher	175(1)			0.9(0.62,1.31)	0.587		
**Mother’s marital Status**							
Married	12,276(70)			1.00			
Living together	2663(15)			1.04(0.94,1.15)	0.456		
Widowed/Divorced/Live Apart	1760(10)			1(0.90,1.13)	0.934		
Never Married	797(5)			0.99(0.84,1.17)	0.891		
**Wealth index**							
Poorest	4132(24)			1.00			
Poorer	3678(21)			0.92(0.83,1.02)	0.12		
Middle	3525(20)			0.84(0.75,0.93)	0.001		
Richer	3334(19)			0.67(0.60,0.76)	<0.001		
Richest	2827(16)			0.55(0.47,0.64)	<0.001		
**Individual characteristics**							
**Age**							
0–5	1856(11)					1.00	
6–11	1910(12)					1.85(0.77,4.45)	0.167
12–23	3718(22)					2.25(0.98,5.17)	0.057
24–35	3235(20)					1.41(0.61,3.23)	0.419
36–47	3030(18)					0.76(0.34,1.71)	0.505
48–59	2828(17)					0.63(0.28,1.45)	0.278
**Sex**							
Female	8707(50)					1.00	
Male	8789(50)					1.12(0.98,1.27)	0.085
**Birthweight**							
Low	1319(13)					1.00	
Normal or High	8714(87)					0.92(0.76,1.13)	0.431
Dietary diversity score *							
Below 3	1690(43)					1.00	
3 and above	2259(57)					0.93(0.76,1.19)	0.568
**Month of Breastfeeding**							
<6	1859(16)					1.00	
6–12	2696(23)					1.8(0.78,4.15)	0.171
13–24	5914(51)					1.23(0.55,2.74)	0.611
>24	828(7)					1.08(0.47,2.45)	0.857
Never breastfed	230(2)					1.51(0.62,3.63)	0.362
**Stunting**							
Normal	9931(61)					1.00	
stunted	6245(39)					1.23(1.06,1.43)	0.007
**Underweight**							
Normal	13,791(85)					1.00	
Underweight	2413(15)					1.05(0.84,1.32)	0.64

N(%): N frequency; (%) percentage of frequency, AOR: Adjusted Odds Ratio, C.I.: Confidence interval. * Dietary diversity data was lacking in 2004-2005 dataset. ^a^ Model-1: adjusted for survey phase. ^b^ Model-2: adjusted for survey phase and all variables shown under Model-2. ^c^ Model-3: adjusted for survey phase and all variables shown under Model-3.3.3. Regional Discrepancy in Child Anemia Trends in Tanzania in the Past One Decade.

**Table 4:** Factors associated with child anemia using TDHS 2015–2016.

Variable	N (%)	Model 1 ^a^		Model 2 ^b^	
		AOR(95% CI)	*p*-Value	AOR(95% CI)	*p*-Value
**Household characteristics**					
**Type of residence**					
Urban	4099 (23)	1.00			
Rural	13,397 (77)	0.87(0.75,1.01)	0.059		
**Family size**					
1–4	4230 (24)	1.00			
5–9	10,078 (58)	1.01(0.89,1.14)	0.910		
10+	3188 (18)	1.15(0.96,1.37)	0.127		
**Number of children under five**					
1	5553 (33)	1.00			
2	6790 (40)	0.99(0.88,1.10)	0.802		
3	2993 (18)	1.16(1.00,1.36)	0.056		
>3	1518 (9)	1.41(1.13,1.76)	0.002		
**Mother’s age at first birth**					
<15	511 (3)	1.00			
15–19	10,609 (61)	0.8(0.59,1.09)	0.152		
20–24	5288 (30)	0.82(0.60,1.11)	0.199		
≥25	1089 (6)	0.68(0.48,0.97)	0.033		
**Mother’s education level**					
No education	4099 (23)	1.00			
Primary	11,668 (67)	0.72(0.64,0.82)	<0.001		
Secondary	1554 (9)	0.84(0.70,1.01)	0.061		
Higher	175 (1)	0.66(0.38,1.12)	0.120		
**Mother’s marital Status**					
Married	12,276 (70)	1.00			
Living together	2663 (15)	1.03(0.92,1.16)	0.600		
Widowed/Divorced/Live Apart	1760 (10)	0.93(0.81,1.08)	0.374		
Never Married	797 (5)	0.92(0.74,1.14)	0.431		
**Wealth index**					
Poorest	4132 (24)	1.00			
Poorer	3678 (21)	0.97(0.85,1.12)	0.685		
Middle	3525 (20)	0.94(0.81,1.09)	0.401		
Richer	3334 (19)	0.71(0.61,0.84)	<0.001		
Richest	2827 (16)	0.66(0.53,0.81)	<0.001		
**Individual characteristics**					
**Age**					
0–5*	1856 (11)				
6–11	1910 (12)			1.00	
12–23	3718 (22)			1.44(0.82,2.53)	0.207
24–35	3235 (20)			0.61(0.19,1.96)	0.402
36–47	3030 (18)			0.38(0.08,1.76)	0.218
48–59	2828 (17)			0.18(0.03,1.03)	0.053
**Sex**					
Female	8707 (50)			1.00	
Male	8789 (50)			1.39(1.10,1.75)	0.005
**Birthweight**					
Low	1319 (13)			1.00	
Normal or High	8714 (87)			0.61(0.40,0.94)	0.026
Dietary diversity score *					
Below 3	1690 (43)			1.00	
3 and above	2259 (57)			0.94(0.78,1.15)	0.458
**Month of Breastfeeding**					
<6	1859 (16)				
6–12	2696 (23)			1.00	
13–24	5914 (51)			0.5(0.29,0.87)	0.014
>24	828 (7)			0.91(0.25,3.32)	0.887
Never breastfed	230 (2)			1.14(0.30,4.41)	0.845
**Stunting**					
Normal	9931 (61)			1.00	
stunted	6245 (39)			1.08(0.81,1.44)	0.612
**Underweight**					
Normal	13,791 (85)			1.00	
Underweight	2413 (15)			0.99(0.65,1.50)	0.953

N(%): N frequency; (%) percentage of frequency, AOR: Adjusted Odds Ratio, C.I.: Confidence interval. *children below 6months had no anemia data in 2015 dataset. ^a^ Model-1: adjusted for survey phase. ^b^ Model-2: adjusted for survey phase and all variables shown under Model-2.
